# Can artificial ecological corridors be used for ecological restoration of cultivated land in Chinese Mollisols?

**DOI:** 10.3389/fpls.2022.977297

**Published:** 2022-09-23

**Authors:** HuiBo Xu, SongTao Wu, Jessica Ann Diehl

**Affiliations:** ^1^ School of Architecture, Harbin Institute of Technology, Harbin, China; ^2^ Key Laboratory of Cold Region Urban and Rural Human Settlement Environment Science and Technology, Ministry of Industry and Information Technology, School of Architecture, Harbin, China; ^3^ School of Design and Environment, National University of Singapore, Singapore, Singapore

**Keywords:** ecosystem sustainability, ecological corridor, mollisols, ecological restoration, soil quality

## Abstract

Artisficial ecological corridors (AECs) are internationally recognized as a standard method for restoring the regional ecological environment. However, the coupling relationship between AECs and soil quality has rarely been studied. Harbin, a typical mollisols region in the cold area of China, has severe soil problems and remediation is urgently needed, yet AEC research in this region is lacking. Based on the perspective of soil restoration, the construction factors of ecological corridors are quantitatively evaluated. It can predict the long-term impact of AECs already built along Harbin’s Ashi River on soil chemical indices. This research studied the ecological restoration of secondary woodland, cultivated land within the ecological corridor, and cultivated land outside the influence range of the corridor under the influence of continuous recovery time and different locations in the corridor (distance from the Ashe River). Soil samples were taken from 5 plots, with a total of 161 samples, and 12 indices of soil ecological characteristics were monitored. The result are as follows: It is believed that the quality restoration of mollisols through ecological corridors has great application potential. Based on the low-cost natural restoration of ecological corridors, the highest values of total phosphorus (TP) and soil organic matter (SOM) in soil indices were detected in corridors (restored for more than 10 years). In addition, after ten years of recovery, pH and electrical conductivity (EC) in the ecological corridor returned to normal from high levels in cultivated land that far exceeded the reference values. The recovery process of mollisols mass begins to decrease, then increases, and finally reaches and exceeds the reference value of standard mollisols. The redundancy analysis of soil samples found the distance to be a key factor affecting soil total nitrogen (TN), SOM, and cation exchange capacity (CEC). Recovery time is a crucial factor affecting soil total organic carbon (SOC), pH and EC. According to the TN, SOM, and CEC mollisols indices, the ecological corridor’s unilateral width is 125-150m. According to the SOC, pH, and EC indices of mollisols, the AECs should complete a natural recovery cycle of a minimum of 13 years. This study reveals the change mechanism of soil quality in mollisols area corridors based on recovery time and location. This research offer ideas and a scientific basis for worldwide governments in mollisols to formulate mollisols restoration policies.

## Introduction

Mollisols cover more than 916 million hectares globally, accounting for 7% of the world’s ice-free land area, 3.2% of the land surface, and 28.6% of cropland across all soil types (Lal et al., 2003; Liu et al., 2013). Mollisols are mainly distributed globally in the United States, Ukraine, China, and Argentina (Xu et al., 2020). These four Mollisols regions together form the natural granary of the world; they are an essential guarantee of world food security. It is critical to understand this soil and its risks. At present, global mollisols are increasingly threatened by human-driven land-use changes, and a large number of the original forest land on mollisols has been converted into cultivated land, where the food production capacity and human pressure on the natural environment are high (Rui et al., 2020; Gibson et al., 2011).

In North America, most of the mollisols area was converted to agricultural land as early as the 19th century (Buyanovsky and Wagner, 2002). Over the last century, unsustainable land use in North America due to cultivated land degradation, soil nutrient imbalances, frequent wind erosion, and other factors has reduced crop yields by 20-40% in mollisols regions (Posner et al., 2008; Xu et al., 2020). Under intensive, mechanized, monocropping, soil quality in North American mollisols regions has declined by more than 50% over the past 100 years (Gollany et al., 2011). According to Lal, 2014 forecast (Lal, 2014), if the degradation of U.S. mollisols continues, grain yields could decline by more than 16.5 per cent by 2020. In China, the loss of mollisols quality is even more severe. In 2019 alone, water erosion of 67,200 square kilometres and wind erosion of 8,300 square kilometres of cultivated land occurred in China’s mollisols belt of China (Ministry of Water Resources of the People's Republic of China, 2019). Water, wind, freeze-thaw and other erosion problems are severe in the mollisols area of China.

The ecological problem of global mollisols is at odds with the implementation of the “Four per Thousand” initiative proposed at the Paris Climate Summit in November-December 2015 (Cornelia et al., 2018). The initiative emphasizes sequestering global carbon into soils at a depth of 40 cm and an ideal rate of 0.4 per cent per year (Lal, 2016). Extensive, human-dominated productive landscapes across the globe could have dire consequences for mollisols ecosystems primary forests of global mollisols are converted into agricultural production lands and cultivated for years (Wilson et al., 2020; Deng et al., 2022).

Human land use and land cover changes make the habitats of organisms in mollisols areas more dispersed. The result is that mollisols ecosystems are further damaged, and the self-stabilization and recovery capabilities of ecosystems are further reduced. Organisms encounter increasing difficulties in population and genetic exchange between small and fragmented suitable habitats (Montoya et al., 2021). Restoration of artificial ecological corridors (AEC) can expand from isolated patches to more suitable animal habitats (Habel et al., 2020), and reduce human-wildlife conflicts and the chance of harm to both. They can promote population growth and flow of animal genes between different patches to restore biodiversity across a region (Hale et al, 2001). An essential means of ecological restoration of natural resources by ecologists worldwide is to establish and maintain a connected network. It is used to maintain the connection of critical ecological patches in the site to alleviate landscape fragmentation, restore biodiversity, and maintain and achieve restoration of natural ecological resources (Su et al., 2021).

Beginning in the 1970s, the Chinese government began to vigorously develop the forest protection forest system in Northeast China. After 45 years, a shelter forest system, or ‘shelterbelt’, of 36.7 million hectares has been formed in the mollisols farming area of Northeast China (Shidong and Degan, 2021). In 2010, the Harbin government started building an ecological corridor system within the city area based on the shelterbelt (Harbin Municipal People’s Government, 2022). Through these projects, the government hopes to achieve and improve the resilience and erosion resistance of the mollisols ecosystem in the region. At the same time, shelterbelts can better provide various ecological services for downstream urban areas (Xu et al., 2022). At present, the shelterbelt system has a large-scale distribution in the mollisols area of Northeast China and has played an essential role in restoring the regional ecological environment. Yet, there is a lack of comprehensive and quantitative evaluation research on the relationship between the construction factors of forest corridors and mollisols quality improvement.

Frigid mollisols soils are more susceptible to poor land management practices than ordinary soil (You et al., 2021). Compared with ordinary soil, mollisols subsoil is heavy and has poor scour resistance, and it is not easy for rainwater to infiltrate. In the rainy season, it is more likely to cause rapid sinking and drainage of water bodies, resulting in soil erosion (Li and Wu, 1989). At the same time, mollisols cultivation involves intensive human activities, resulting in a decrease in the pH value and organic matter content (Wilson et al., 2020). Mollisols are also important soils in pasture and pasture systems worldwide. They are prone to soil erosion, de-corruption (loss of stable aggregates and organic matter), and suffer from anthropogenic soil acidity (Liu et al., 2012). In addition, the freeze-thaw cycle erosion unique to the cold mollisols belt is also considered the critical factor in inducing and accelerating the erosion trench. These reasons together increase the erosion of the chernozem soil (Zhao et al., 2012). This degradation phenomenon is one of the most severe and directly leads to the massive loss of mollisols and the inoperability of agricultural machinery (Xu et al., 2019).

Given these conditions, the objective of this research is 1. understanding the long-term impact of the ecological corridor projects on restoring mollisols’ properties and functions in cold regions using the example of Harbin. 2. determining the forest-river ecological corridor’s most effective width. 3. determining the forest-river ecological corridor’s most effective recovery time. We investigated a forest-river ecological corridor (Ashi River) completed in Harbin. Along the Ashi River ecological corridor, the construction time of each section varied. We believe this research provides a reliable baseline assessment for soil quality and ecosystem restoration. It can inform local governments in formulating land use planning and ecological network designs that benefit soil ecosystems.

## Materials and methods

### Study site

**Figure 1:**
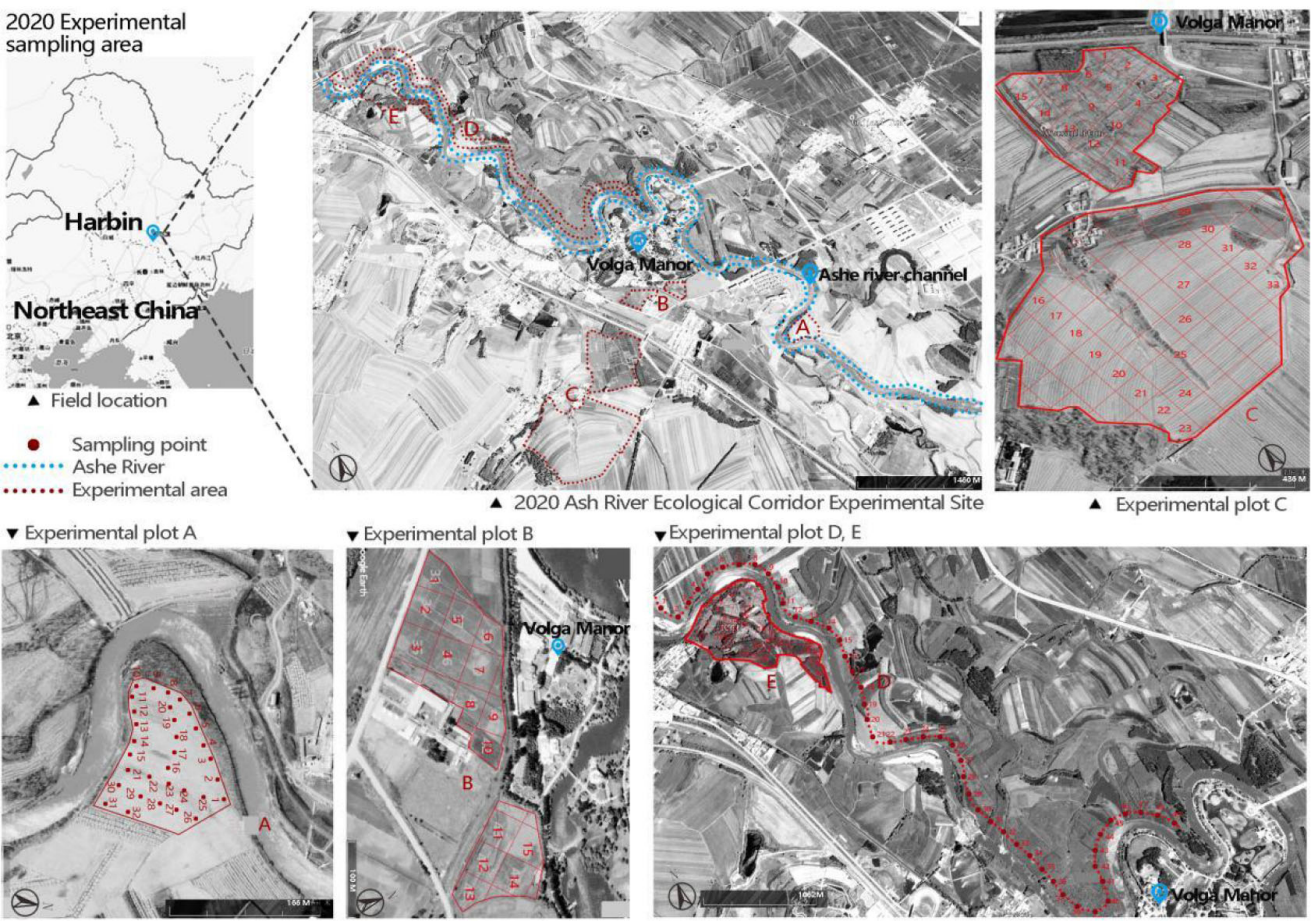
Distribution of 161 sampling points in the experimental area **(A–E)**. (The range map of the Northeast China comes from Xu et al, 2022).

The study was conducted in the Volga Manor section of the Ashe River Ecological Corridor on the outskirts of Harbin (125°42′~130°10′ E, 44°04′~46°40′ N). Harbin is located in the central part of Heilongjiang Province and has a temperate monsoon climate (**Figure 1A**). The average annual precipitation is 569.1 mm, concentrated in June-September. Summer accounts for 60% of the annual precipitation. The concentrated snowfall period is from November to January of the following year. There are four distinct seasons. The average temperature in January is -19°C; the average temperature in July is 23°C (weather data comes from the Harbin Weather Station).

The experimental design consisted of five sample plots. Three plots were reference plots: Plot a randomly arranged 32 plots as a reference restoration type (within the ecological corridor influence range 0 m < Distance from the center of the ecological corridor < 300 m (Yuwu et al., 2013); continuous planting of maize). Plot b was divided into 15 sample points using the grid method as a reference restoration type (within the ecological corridor’s influence area; continuous rice cultivation). Plot c was divided into 33 sample points using the grid method as the initial restoration type (outside the ecological corridor’s influence range; continuous planting of rice and corn). Two plots were restoration plots: Plot D was arranged with a sample point every 50 m and a total of 49 sample points (between 30-120 m from the Ash River channel). Plot E used the grid method for 32 sample points (the recovery time was between 7-and 35 years) (**Figure 1**). All sample points were located in the typical mollisols belt at an altitude of 126.00 - 128.25 m, and the soil type was chernozem. The sample plots had been cultivated for more than 70 years. Compared with ordinary chernozem soil, the chemical indices of the cultivated mollisols had experienced soil degradation, including soil salinization and compaction (Ding, 2003).

**Figure 2:**
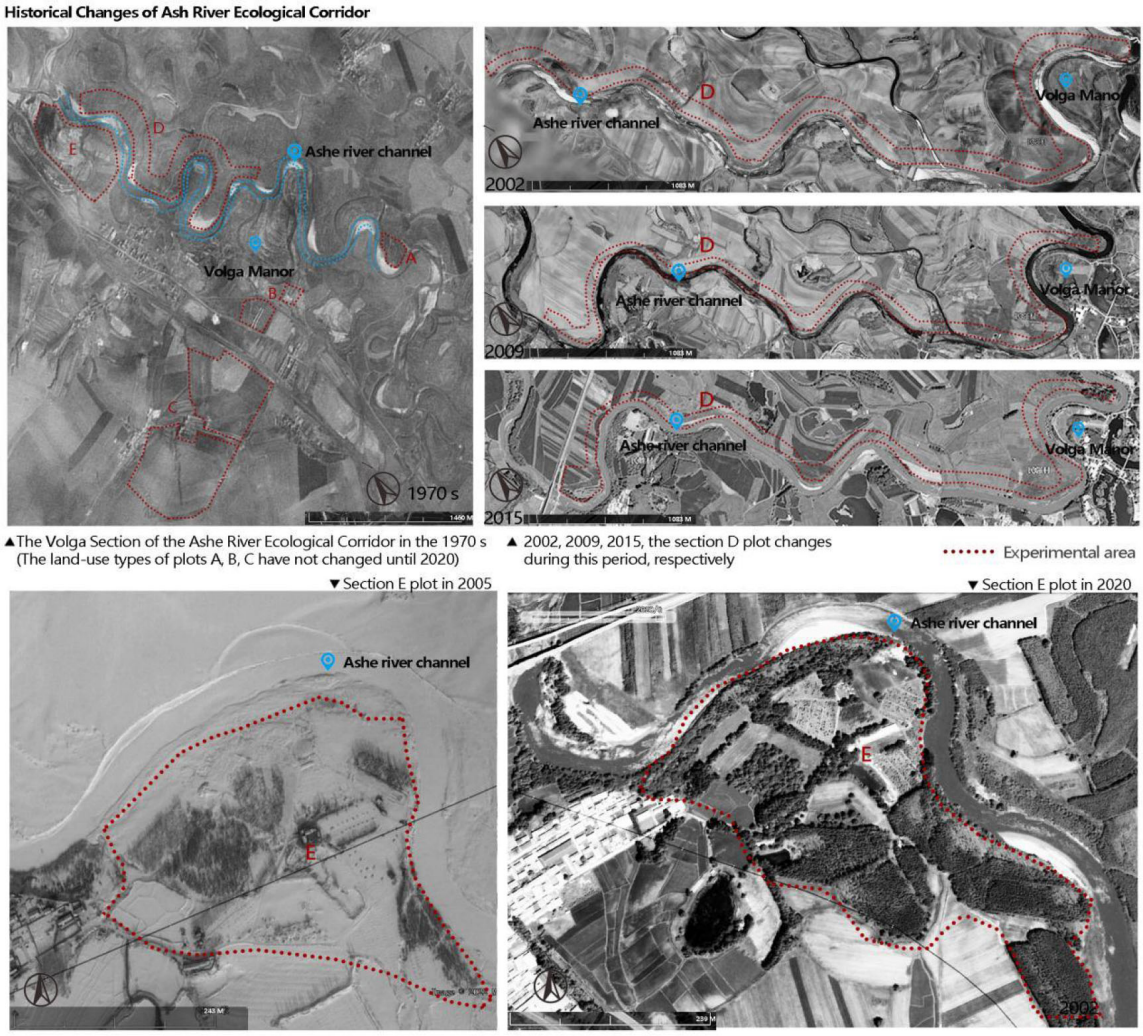
The number of years of recovery for all samples studied was based on local maps, historical archives, aerial photographs, and field surveys. (Photographs from USGS and CNES/AIRBUS) The small characters in the above picture show the location and shooting date of each picture sampling area.

Using historical data, United States Geological Survey’s maps (1970), and aerial imagery from 1985 to 2021 (Imagine@CNES/AIRBUS), we constructed the recovery time series of the ecological corridors from 0 to 35 years (see Jonathan Lenoir’s research for details on methods for determining recovery times (JLenoir et al., 2021)). For ecological corridors that appeared between two consecutive historical maps, we took the intermediate dates of the two aerial images and combined the survey method of plant age in the field and consultation with farmers to pinpoint the time of corridor restoration. Based on available images, historical data, and field investigations, we determined the recovery time of soil samples (**Figure 2**; **Table 1**).

**Table 1:** Essential characteristics of the five study plots.

Vegetation Features	A	B	C	D	E
Recovery time	Initiated (Figure 2)	Initiated (Figure 2)	Initiated (Figure 2)	2-35 years (Figure 2)	7-35 years (Figure 2)
Dominant plant	Corn	Rice	Rice, Corn	Elm*(Ulmus pumila Linn.)*, popla*(Populus tomentosa Carr.)*, Forsythia*(Forsythia suspensa (Thunb. Vahl)*, Plum tree*(Amygdalus triloba (Lindl.) Ricker)*	Elm*(Ulmus pumila Linn.)*, popla*(Populus tomentosa Carr.)*, Forsythia*(Forsythia suspensa (Thunb. Vahl)*, Plum tree*(Amygdalus triloba (Lindl.) Ricker)*
Plant management	Fertilization 2 times yr^−1^; mixed fertilizer (N:P:K=4:3:2) 180 kg ha^−1^yr^−1^;	Fertilization 2 times yr^−1^; mixed fertilizer (N:P:K=4:3:2) 180 kg ha^−1^yr^−1^;	Fertilization 2 times yr^−1^; mixed fertilizer (N:P:K=4:3:2) 180 kg ha^−1^yr^−1^;	NO	NO
Historical land use type	cultivated land	cultivated land	cultivated land	Cultivated land, gradually transformed into artificial ecological corridors	Cultivated land, gradually transformed into artificial ecological corridors

### Soil sampling and analysis

Soil sampling was carried out in November 2020 after the Northeast China plant growing season had ended and the cultivated land had been harvested. For Plot A, we used random sampling. In Plot D, we used the interval distribution method. In Plots B, C, and E, we established a 50 m × 50 m survey grid. With each grid centre as the centre of the triangle, we took three vertices and centre points of the equilateral triangle with an edge length of 30 m, four points. After carefully removing the litter and grass layer, we used the soil spiral drill to take four composite soil samples at 0-20 cm depth. After removing visible plant debris (including roots and stones), we mixed them into one example (about 500 g). One soil sample (250 g) was screened with a 2 mm sieve and kept at 4°C and the biochemical analysis was performed in less than two weeks. Another soil sample (250 g) was dried for about two weeks at room temperature (~ 20°C) and stored in a closed container for chemical analysis.

According to the soil nutrient standard of China’s ‘Second China Soil Survey’, Singapore’s ‘General Landscape Soil Mixture Standard’ (CUGE (Centre for Urban Greenery and Ecology, 2013), which is widely used internationally, and the United States ‘Landscape Topsoil Standard’ (ASTM D5258-13), we selected ten ecological indices commonly used to measure soil quality comprehensively. These included: mollisols thickness, pH, conductivity, cation exchange capacity, soil total organic carbon, soil organic matter, dry matter content, total nitrogen, total phosphorus, and total potassium. We measured variables according to the method in **Table 2**.

**Table 2:** Analysis method of soil variables.

Soil variable	Abbreviation	Method	Reference
Soil thickness	ST	/	/
pH	pH	pH-meter method (soil: water ratio 1:2.5)	(Page et al., 1982)
Electrical conductivity	EC	Conductivity method (soil: water ratio 1:5)	(Page et al., 1982)
Cation exchange capacity	CEC	Hexamminecobalt trichloride solution-Spectrophotometric method	(Ranger et al., 1990)
Soil total organic carbon	SOC	Wet digestion method	(Nelson and Sommer, 1975)
Soil organic matter	SOM	Potassium dichromate volumetric method	(Claudio et al., 1991)
Dry matter content	DMC	Time Domain Reflection method	
Total nitrogen	TN	Kjeldahl method	(Bremner and Mulvaney, 1982)
Total phosphorus	TP	Mo-Sb colorimetric method (Involving digestion of soil samples with perchloric acid)	(Yuan and Lavkulich, 1995)
Total potassium	TK	Flame spectrometry method (Involving preliminary nitric acid digestion followed by a hydrochloric acid solubilization)	(Silva et al., 1999)

### Data analysis

We first used the Origin box diagram to exclude outliers from the collected sample data. We used SPSS V22.0 for Windows software for a single-factor analysis of variance to compare the chemical indices of different soils including land types (cultivated land, artificial ecological corridors), restoration years (0-35 years), and different distances from the ecological corridor centre (10-300 m). We used redundancy analysis (RDA) to demonstrate the critical ecological corridor construction indices affecting soil environmental stoichiometry. The RDA allows correlation analysis of multiple dependent variables (CEC, EC, SOC and SOM) on multiple independent variables (distance and recovery time). Finally, a linear model was estimated to select the most suitable ecological corridor construction indices. We performed all statistical tests at a 0.05 significance level and visualised data through Origin 2020 and R. In terms of control variables and analysis, we limited and extracted the sampling data according to the different sections of the problem (**Table 3**).

**Table 3:** Screening of raw data.

Chapter serial number	Data selecting	Contain sample plots	Cause
3.1	We selected the ecological corridor from the river distance > 10 m sampling points, an ecological corridor outside the scope of cultivated land (in Harbin’s typical mollisols zone, from the Ash River > 300 m).	C, D, E	Few natural factors affect the quality of mollisols when the sampling points with a distance > 10 m are affected by uncontrollable factors such as river erosion
3.2 **Figure 3**	We selected the recovery time as relatively particular (30-35years), different distance data (distance > 10m).	D, E	Control variables to improve experimental accuracy
3.2 **Figure 4**	We selected a relatively certain distance (distance 70 m-100 m) and different recovery time data.	D, E	Control variables to improve experimental accuracy
3.3 **Figure 5**	We selected samples (10m < distance < 300m) of cultivated land and forest affected by ecological corridors.	A, B, D, E	Comprehensive analysis of the influence of ecological corridor construction factors on soil within the scope of influence
3.3 **Figure 6**	We selected samples from stable ecological corridors (Distance from the Ash River > 30 m, recovery time > 5 years)	D, E	Control variables to improve experimental accuracy
3.4 **Figure 7**	We select the samples in the ecological corridor that were stable and affected by the ecological corridor distance (distance from the ecological corridor > 30 m, 35 a ≥ recovery time ≥ 20 a)	D, E	Control variables to improve experimental accuracy
3.4 **Figure 8**	We selected samples (100m ≥ Distance from Ash River ≥ 45m, recovery time > 3 years) which were affected by the recovery time of the ecological corridor.	D, E	Control variables to improve experimental accuracy
3.5	We limit the distance between the sampling points and the Ash River (30-80m). In addition, we introduced cultivated land samples outside the ecological corridor (distance > 300 m) and continuously cultivated.	C, D, E	Control variables to improve experimental accuracy Plot C is introduced to simulate the restoration of cultivated land in 0 years to make the time series more coherent.

## Results

### Difference between ecological corridor and cultivated land

Of the 10 soil properties, 7 (~70%) showed overall differences between forest-river ecological corridors and cultivated land (**Table 1**). Among soil acid-base indices, the pH and EC of cultivated land and corridors restored for more than 10 years differed significantly. Compared with cultivated land, the pH and EC of ecological corridors recovered over 10 years were reduced by 13% and nearly 64%, respectively (**Table 1**). Among soil carbon-related variables, there was no significant difference between cultivated land and ecological corridors that had been restored for more than 10 years. However, the SOM of ecological corridors restored for more than 10 years increased by 19.9% compared with cultivated land. Among soil physical indices, there were significant differences in Dry matter content between cultivated land and ecological corridors restored for more than 10 years, while soil ST did not differ significantly between the two. There was a significant difference in CEC between cultivated land and ecological corridors restored for more than 10 years, and the CEC of the original cultivated soil decreased by about 36%. Compared with the TP of the two, the TP in the ecological corridor restored for more than 10 years increased by about 66%. There were no significant differences in soil TN and TK (**Table 4**). Compared with the 1-5 years corridor, the 5 indices (~50%) in the original arable soil decreased significantly, including ST, pH, EC, CEC, and SOC. Two indices (~20%) in the original cultivated land increased significantly, including DMC and TP. There were no significant differences in soil SOM, TN or TK (**Table 4**).

**Table 4:** Mollisols and site properties for the ecological corridor, cultivated land, standard deviation in brackets.

indices	Unit	cultivated land (Outside the ecological corridor)	Ecological Corridor(1-5a)	Ecological Corridor(>10a)	Reference Value
ST	cm	85.03(24.72)a	67.64(16.23)b	75.23(19.17)ab	60~80~100 (Xiuwen, 2002; Wei et al., 2003)
pH	/	7.44(0.78)a	6.08(0.53)b	6.47(0.22)b	5~7 (Chi et al., 2018)
EC	ds m^-1^	0.16(0.06)a	0.06(0.03)b	0.06(0.02)b	0.05~0.06 (Dun et al., 2017)
CEC	(cmol (+) kg^-1^)	25.31(5.11)a	13.89(3.54)b	16.13(5.87)b	15~20 (China Agricultural Encyclopedia Editorial Committee Veterinary Volume Editorial Committee (CAEECVVEC), 1996)
DMC	%	94.63(1.37)b	97.57(0.94)a	97.39(1.58)a	97.23~98.23 (Wang et al., 2020)
SOC	g kg^-1^	21.15(5.868)a	12.24(3.525)b	17.77(6.77)a	14.43 (Fang et al., 2003)
SOM	g kg^-1^	35.34(13.31)	34.88(11.77)	42.40(12.28)	19.5 (Rui et al., 2020)
TN	mg kg^-1^	1437.61(365.19)	1073.55(518.11)	1305.74(657.88)	/
TK	%	2.27(0.07)	2.26(0.12)	2.29(0.10)	/
TP	%	0.042(0.008)b	0.068(0.010)a	0.070(0.011)a	0.06~0.08 (Li et al., 2021)

CEC = Cation Exchange Capacity by ammonium acetate in pH 7.

The experimental site belongs to the soil-forming range of black calcium soil in Northeast China, and the reference value of the above table is the normal black calcium soil value.

Sampling depth limited to topsoil (root-dense area). ower case letters indicate significant differences between the sites (α= 0.05).

### Ecological corridor construction factors affecting mollisols ecochemical indices

**Figure 3:**
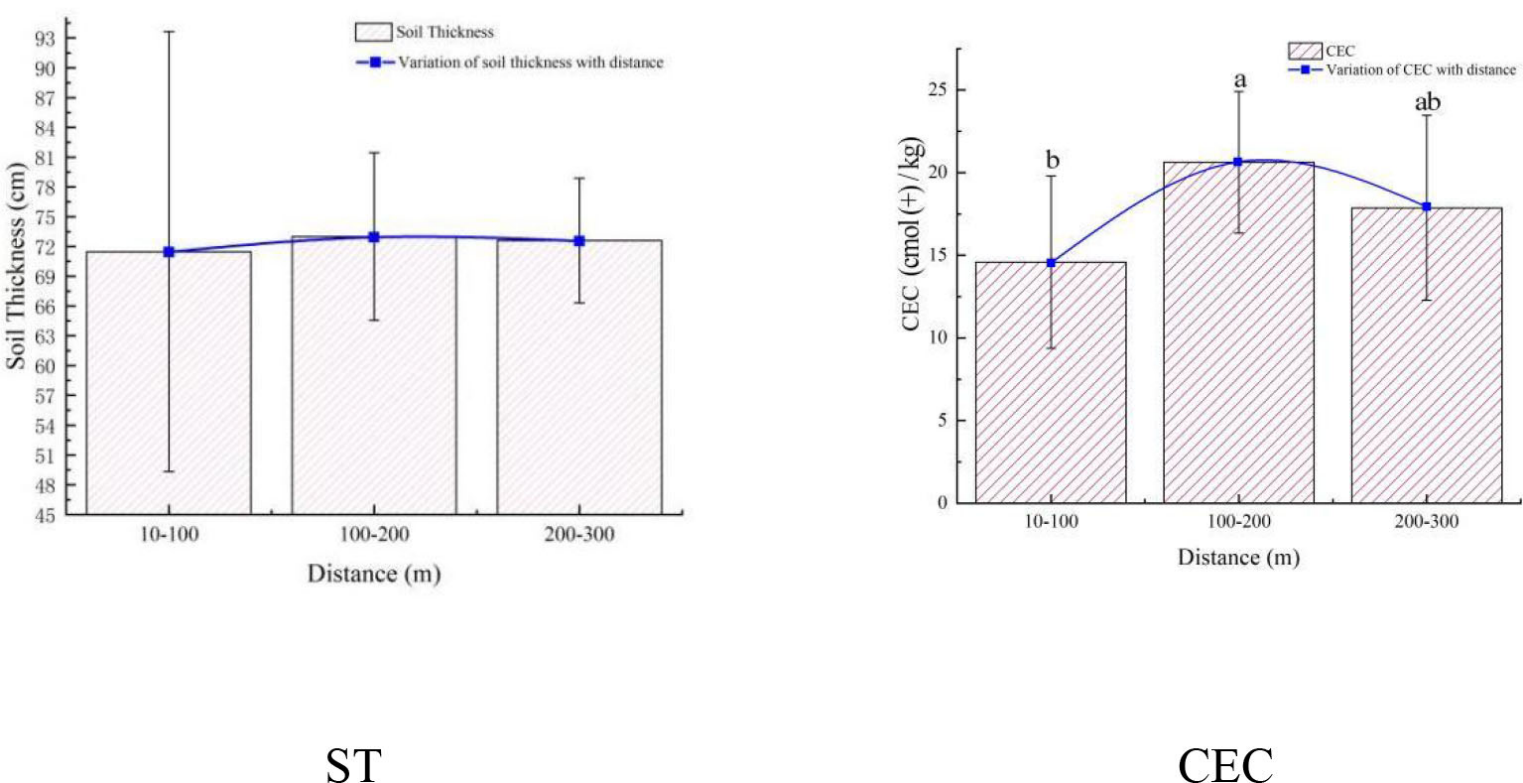
Changes in soil indices with different distances. The error rod is ± standard error.

The analysis results show the restoration effect within the ecological corridor based on the distance from the Ashe River (**Figure 3**). There was no significant difference in the ST indices with the distance in the ecological corridor. However, we found that ST changed with distance from low to high and then lower again. The averages of the three data groups are 71.47cm -73.00cm -72.60cm, respectively. It exhibits a maximum value in the range of 100-200m. In addition, the standard deviation is the largest in the field of 10-100m, indicating that the ST indices of mollisols had firm heterogeneity in this range. The CEC indices varies significantly with the distance. We found that the change of CEC with distance showed the characteristics of low to high and then lower, and the average values of the three sets of data were 14.58Ufr-20.63Ufr-17.87Ufr, respectively. It exhibits a maximum value in the range of 100-200m.

**Figure 4:**
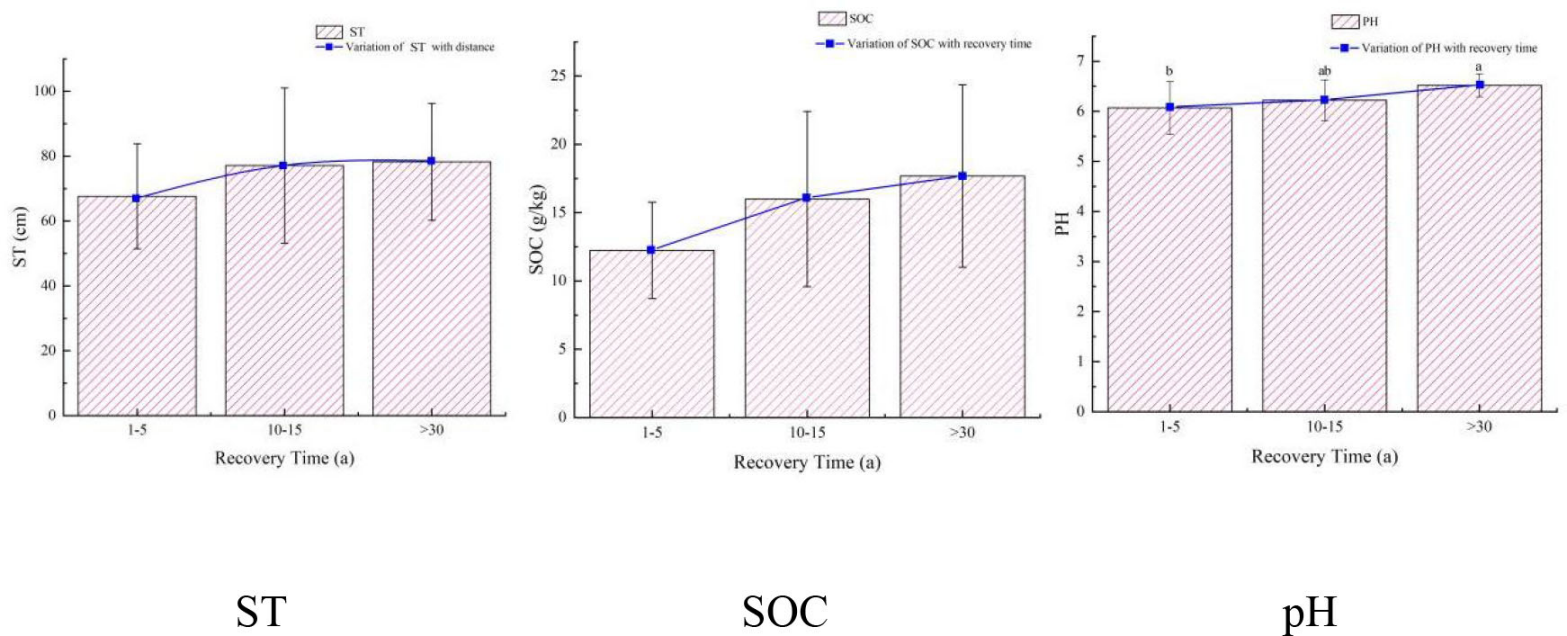
Changes in soil indices with different recovery time. The error rod is ± standard error.

The impact of the recovery time of the ecological corridor on the restoration effect is shown in **Figure 4**. The ST indices increased within the ecological corridor with the recovery time, and there was no significant difference. However, we found that ST increased with the recovery time. The average values of the three groups of data were 67.64cm-77.13cm-78.25cm. From 1-5 years to 10-15 years, the change in the three data sets was the largest, increasing by 14%. There was no significant difference in the SOC indices with the increase in recovery time. Coincidentally, the growth of the SOC indices with the recovery time showed a trend more consistent with the ST indices. Also, from 1-5 years to 10-15 years, the increase in the three data sets was the largest, reaching 30.6%. The pH indices increased with the recovery time, and the difference was significant. We found that the pH indices showed a relatively consistent trend with the ST indices with the increase in recovery time. The average values of the three data groups were 6.07-6.22-6.52, with a slight change in the rise.

### Effects of constructive factors on soil ecochemical indices in ecological corridor

**Figure 5:**
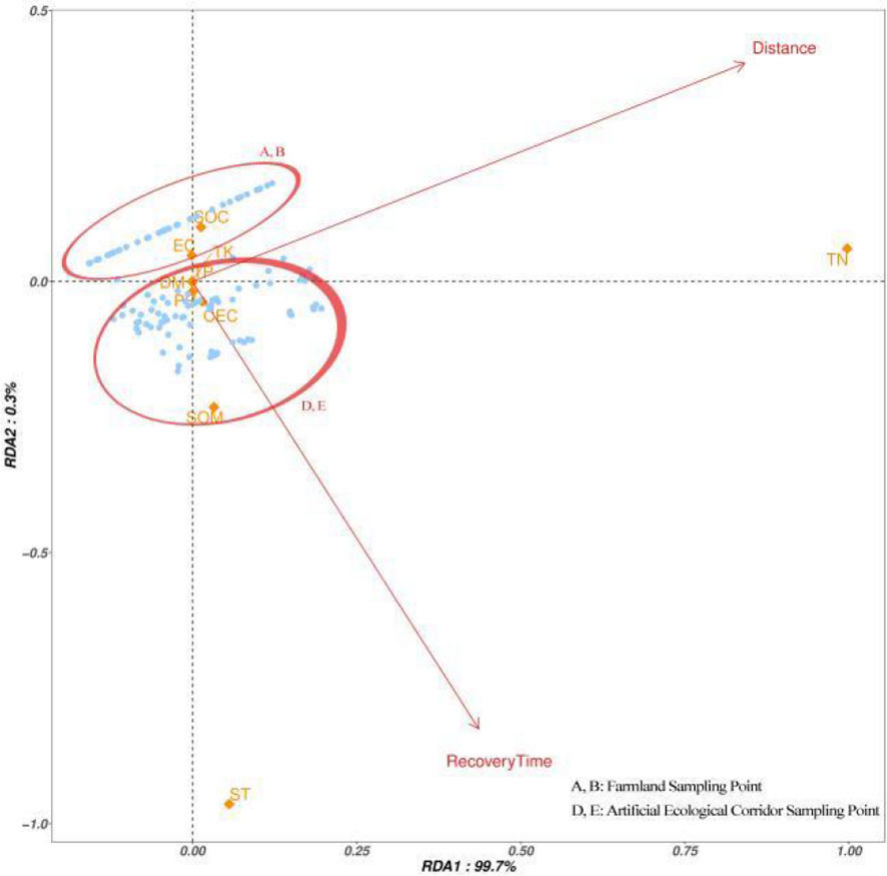
Cultivated land within 300 m from the ecological corridor and all collected samples in the ecological corridor.

**Figure 6:**
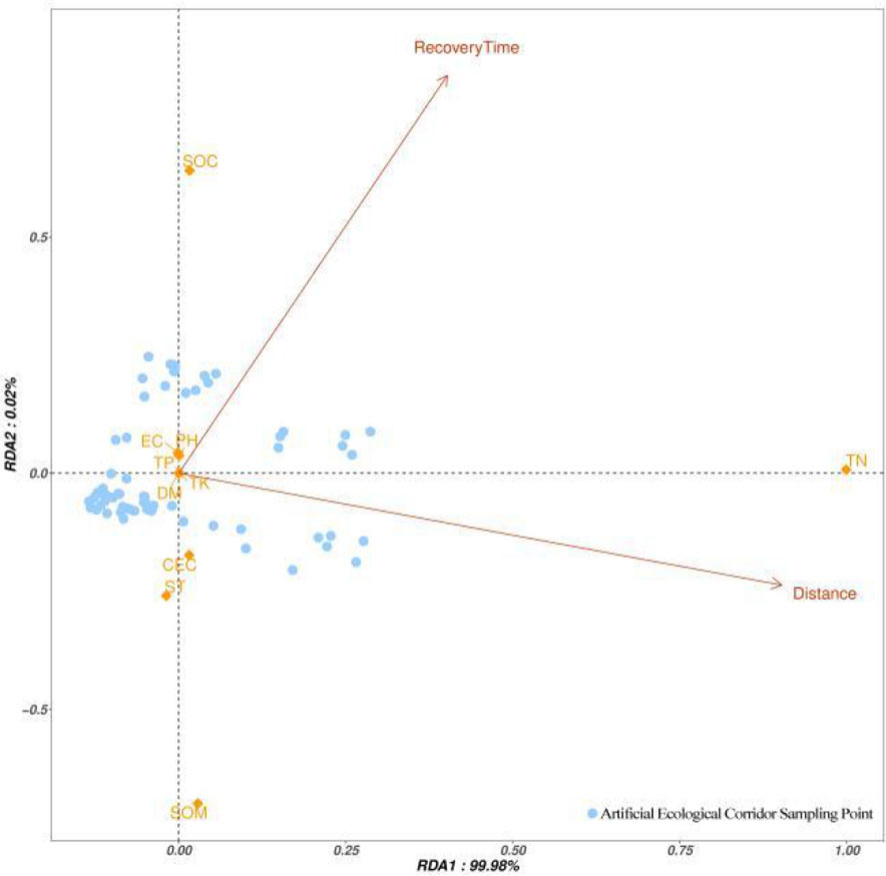
The samples collected within the ecological corridor distance from the Ashe River> 30 m and recovery time > 5 a.

In an RDA plot, the cosine of the arrow angle approximates the correlation between variables: Angles close to 90 degrees indicate less correlation between variables, angles away from 90 degrees and close to 0 degree or 180 degrees indicate a strong positive or negative correlation. We found that there were differences in the distribution of the sample points (A, B) in the cultivated land and the sample points (D, E) in the ecological corridor, and the distribution of the points in the cultivated land (A, B) was relatively dense and uniform (**Figure 5**). The distribution of A and B shows that the mass distribution among the samples was uniform. The distribution of points in the ecological corridor was large and scattered, reflecting the quality difference between the sampling points, indicating that compared with the cultivated land, the ecological corridor provided a variety of environments for animals to inhabit. Most of the sampling points in the ecological corridors (D, E) in **Figure 5** were affected by the distance from the Ashe River and the recovery time. The recovery time axis for most points had a much smaller angle than the distance axis, which indicates that the quality was mainly affected by recovery time (**Figure 6**). In **Figure 6**, various indices of soil SOC, pH, EC, and TP indices were greatly affected by the recovery time, whereas the TN, SOM, and CEC indices were more affected by the distance from the Ashe River. Comparing the changes in **Figures 5**, **6**, we found that the removal of human-disturbed cultivated land and unstable samples in the ecological corridor enhanced the correlation between SOC and EC in soil chemical indices and recovery time; it improved the correlation between SOM and the distance from the Ash River.

In addition, in the lower-left corners of **Figures 5**, **6**, some sample points are negatively correlated with the recovery time and the distance from the Ashe River. Considering that the sampling area is plain, the elevation of the sampling point had almost no change. This phenomenon may be due to the current construction of ecological corridors, which did not prohibit the entry of people and livestock, and those activities may have affected the distribution of soil quality on the site.

### Influence range and recovery time of forest-river ecological corridor

**Figure 7:**
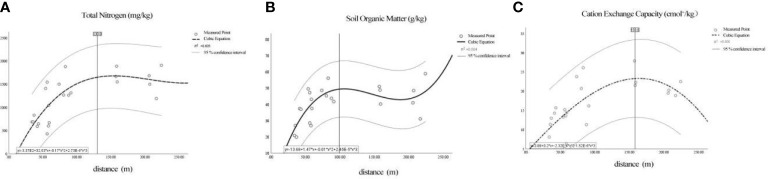
Fit image of ecological chemical indices and distance. The regression function’s upper and lower dashed lines are the 95% confidence intervals of the regression function, including all measurement points. **(A–C)** are the regression images of TN, SOM, CEC (respectively) and distance.

Scope of influence of the forest-river ecological corridor (The optimum width of ecological corridors), we extracted the top three chemical indices in the RDA analysis that were most affected by the distance from the Ash River channel (TN, SOM, and CEC) and constructed models. **Figure 7A** shows the relationship between distance and TN: initially, as the distance increases, the TN content increases. Fitted model: y = -336.66 + 32.63x - 0.169x^2^ + 0.000273x^3^, (r^2^ = 0.605; p = 0.0004), In the samples, after the protection width of the corridor reached 130m, increasing the width slowed down the improvement of the soil TN indices. **Figure 7B** shows the relationship between distance and SOM: initially, the SOM content increased with increasing distance. Fit the model: y = -13.675 + 1.466x - 0.11x^2^ + 0.000024x^3^, (r^2^ = 0.504, p = 0.003). In the samples, after the protection width of the corridor reached 100m, increasing the width slowed down the improvement of the soil SOM indices. However, after the width reached 200m, the soil SOM indices increased to a certain extent with the width increase. **Figure 7C** shows the relationship between distance and CEC: the CEC content first increased and then decreased as the distance increased. Fit the model: y = 3.893 + 0.198 x - 0.00023x^2^ - 0.000002x^3^, (r^2^ = 0.500, p = 0.004). In the samples, when the protection width increased (reaching up to 160m), the soil CEC indices decreased.

**Figure 8:**
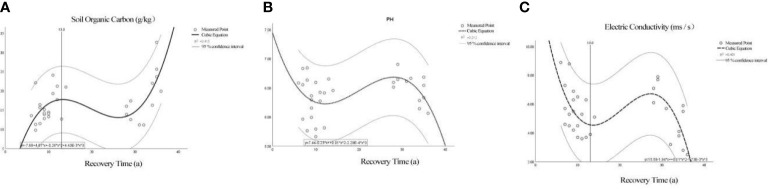
The fitting image of ecological chemical indices and recovery time. The imaginary line is a 95% confidence interval for the regression function, and all measured points are included. **(A–C)** are the regression images of SOC, pH, EC (respectively) and recovery time.

To construct the model for the recovery time of the forest-river ecological corridor, we extracted the top three chemical indices in the RDA analysis that were most affected by the distance from the Ash River: SOC, pH, and EC. **Figure 8A** shows the relationship between recovery time and SOC: initially, as the recovery time increased, the SOC content increased. Fit model: y = -7.683 + 4.610x - 0.262x^2^ + 0.0044x^3^, r^2^ = 0.415, p = 0.002, but after the recovery time reached 13 years, the increased soil SOC indices slowed down or even decreased. **Figure 8B** shows the relationship between recovery time and pH: soil pH first decreased and then increased with the increase of distance. Fit the model: y = 7.438 - 0.234x + 0.0138x^2^ + -0.00023x^3^, r^2^ = 0.212, p = 0.078, The pH values in the collected samples were all within the normal range of weakly acidic mollisols. The regression trend shows that in restoring cultivated land to an artificial ecological corridor, the pH value first decreased, then slowly increased, and finally decreased. **Figure 8C** shows the relationship between recovery time and EC: the EC content first decreases and then increases as the distance increases. Fit the model:y = 15.576 - 1.935x + 0.106x^2^ - 0.0017x^3^, r^2^ = 0.401, p = 0.002, After being restored to artificial ecological corridors in the collected samples, the soil salinity level decreased significantly, which was the same as the pH trend. At about 13 years, the soil EC decreased to the lowest point, and then the soil EC rose slowly, began to decrease again at about 30 years, and finally maintained a lower soil EC value.

### The restoration process of mollisols through an ecological corridor

We selected SOC, pH, EC, and TP because they were significantly correlated with recovery time in RDA analysis. In the first five years of restoration as an ecological corridor, 3 of the 4 soil indices decreased significantly (~ 75%): SOC decreased by nearly 41%, and EC and pH decreased by nearly 65% and 19%, respectively (**Table 5**). The decrease in EC was the largest. Soil SOC increased by about 30% during the 5-10 years of restoration as an ecological corridor. After that, the soil SOC tended to stabilize at around 16 g kg^-1^. Soil pH and EC increased by about 5% and 19%, respectively, during the 5-10 years of restoration as ecological corridors. After that, soil pH and EC tended to stabilize around 6.3 and 5.3Ufr, respectively. In addition, soil TP increased by about 71% in the first five years of restoration as an ecological corridor, after which soil TP stabilized at around 0.07%. The minimum pH value appeared 1-5a after the restoration to the ecological corridor, and it remained stable near the typical value after that time. The minimum values of SOC and EC appeared 15-20 years after the restoration of the ecological corridor. It is related to the lack of data collected during this period (we collected only four sample points in this interval). The second smallest value of SOC appeared in 1-5 years. After becoming an ecological corridor and reducing manual intervention, the content of SOC in mollisols decreased. The minimum value of TP appears to have a recovery time of 0 years. After being restored to an ecological corridor, the TP indices increased and stabilized near the common value.

**Table 5:** Recovery process of mollisols after transforming into ecological corridor.

indices	Cultivated Land(0a)	Ecological Corridor (1-5a)	Ecological Corridor(5-10a)	Ecological Corridor(10-15a)	Ecological Corridor(15-20a)	Ecological Corridor(>20a)
SOC (g kg^-1^)	21.15 (5.868) a	12.42 (3.483) b	16.11 (5.293) ab	17.27 (5.007) ab	9.84 (1.128) b	16.53 (7.096) ab
pH	7.4423 (0.781) a	6.0543 (0.551) b	6.3548 (0.434) b	6.2950 (0.354) b	6.4000 (0.226) b	6.5636 (0.216) ab
EC (ds m^-1^)	0.1567 (0.0626) a	0.0556 (0.03261) b	0.0664 (0.02797) b	0.05 (0.01449) b	0.038 (0.01980) b	0.0531 (0.01902) b
TP (%)	0.042 (0.008) b	0.072 (0.07) a	0.071 (0.07) a	0.070 (0.07) a	0.065 (0.07) a	0.070 (0.07) a

Standard deviation in brackets. Lower case letters indicate significant differences between the sites (α= 0.05).

## Discussion

The research shows that ecological restoration of mollisols can be carried out through ecological corridors. In addition, our results demonstrate that when restoring mollisols through ecological corridors, the quality of mollisols first decreases, then increases, and finally reaches and exceeds the reference value of standard mollisols. According to the TN, SOM, and CEC indices of mollisols, we should keep the recommended unilateral width at 125-150 m, which positively affects the restoration of soil eco-chemical indices. According to the SOC, pH, and EC indices of mollisols, it is suggested that the ecological corridor should complete a natural restoration cycle of at least 13 years.

### Potential of ecological restoration of mollisols in Northeast China by using ecological corridors

The soil in the ecological corridor restored for more than 10 years post-cultivation had similar ST and SOC indices to the cultivated land outside the ecological corridor. It shows that after 10 years of restoration as an ecological corridor, it has initially reached the level of cultivated land. However, the CEC has not recovered to the cultivated land CEC value. It indicated that soil CEC took a longer time (more than 10 years) to restore soil to the level when it was cultivated. The pH and EC reached reference values. It shows that the ecological corridor can restore the seriously acidified soil in the cultivated land to the level of ordinary mollisols. Under the production state of high maintenance and high input nutrients at the time of sampling, the cultivated land outside the corridor’s fertility indices were better than the reference values.

After restoring cultivated land into a forest-river ecological corridor, the soil in the ecological corridor that had been restored for more than 10 years had restored to the level of cultivated land. Part of the indices in the ecological corridor exceeded the level of cultivated land. It is consistent with what was observed in previous studies (Di et al., 2020). The reasons are as follows: 1) The feasibility of soil restoration through the ecological corridor, that is, soil nutrient absorption and accumulation process are easily affected by land-use change and interference’ (Piccolo et al., 1994). 2) The land use mechanism and plants at different sampling points may affect the accumulation of soil nutrients. Different land-use patterns may affect the accumulation pattern of nutrients in the soil ecosystem by affecting the growth rate of plants, the distribution pattern of soil nutrients and the quality of soil nutrients (Lugo, 1992; Hobbie, 1996). 3) The accumulation of soil nutrients results from the mutual exchange and accumulation of soil, plants and animals in the soil ecosystem (Mosier et al., 2021).

In addition, the EC of mollisols in the ecological corridor dropped from the high acid-base value when cultivated land to the expected value after restoration to the ecological corridor. The main sources of salts in cultivated land of mollisols are: 1) Extensive use of chemical fertilizers and pesticides. 2) The farming behavior of local farmers (flooding with flood water) keeps the groundwater at a high level. After the topsoil water evaporates, the salinity rises and accumulates on the surface. 3) The urban water consumption in the upper reaches is large. The rivers in the mollisols belt (such as the Ash River in the experimental area) often have internal interruptions. The salt cannot be transferred and deposited in the mollisols area (Herbert et al., 2015; Wang et al., 2022). After returning farmland to ecological corridors, the EC returned to normal values. The possible reasons are: 1) After the ecological restoration project, the natural rivers in the Ash River Basin have been reflowed. The accumulated water and salt can be discharged in time, and the groundwater level has dropped. 2) After changing the type of land use, the reduction of fertilization amount, the high biological content inside the soil, the reduction of crops that absorb much water in the growing season, and the reduction of plant litter in the initial stage of the ecological corridor. They make the surface soil frequently washed by rainwater, increase water content inside the soil, and salts are transported (Ding et al., 2019).

Previous studies have shown that according to the 10,583 sampling points Yao et al. (Yao et al., 2020) in 2008 from the northeastern mollisols belt, the soil quality in the area where this experiment is located are generally low (grade 7-8). However, according to the Chinese cultivated land quality grading standard (National Technical Committee for Soil Quality Standardization (NTCSQ), 2016) (consistent with Yao’s standard), we found that the ecological corridor in the sampling area belongs to high-quality mollisols (grade 1-2). It may be since Yao’s experiment performed large-scale sampling with low precision, and only a large-scale estimation was carried out for this experimental area. The average interpolation accuracy of different soil properties caused by sampling at different scales varies greatly (Yuan et al., 2022). In addition, this may also be related to the restoration of soil quality along the corridor in the experimental area after nearly 30 years of ecological restoration in the Ashe River Ecological Corridor, showing that the soil quality is better than that of the same soil in the surrounding soil.

However, this also explains the limitations of this study to a certain extent. In the next step, we plan to conduct a larger-scale collection and comparison of cultivated land and corridor soil samples in the area where the Ash River Forest-River Ecological Corridor is located. We want to confirm the feasibility of the artificial ecological corridor for mollisols restoration on a larger scale.

### Recovery process of mollisols by ecological corridor

The research results show that the indices change mainly occurred in SOC, pH, EC and TP indices after cultivated land was restored to the forest-river ecological corridor. The indices decreased significantly in the first 5 years of recovery compared with cultivated land. After the recovery time > 20 years, SOC reached the level of cultivated land, and EC and pH decreased to the average value range. It indicates that ecological corridor restoration of mollisols based is a process of first decreasing and then increasing. Our observations are consistent with Yu’s work (2020), which suggests that the use of ecological corridors for ecological restoration of damaged cultivated land is a long-term process that may lead to carbon release in the first five years. Studies in other regions also show that “soil quality declines when cultivated land is converted to the artificial secondary forest” (Nakvasina and Shumilova, 2021).

Possible reasons for this long-term process are, first, the artificial input of fertilizers on the mollisols is reduced after the cultivated land is transformed into an ecological corridor. Second, in the initial artificial ecological corridor, few animals move in. In addition, fast-growing plants (e.g., poplar, elm) are planted in the growth phase in carrying out restoration of artificial ecological corridors in most areas of China (including our experimental area). In the early stage of fast-growing plants, these trees absorb high levels of soil nutrients, but the plants have little litter to draw from and consume nutrients from the soil (Rancourt et al., 2015). A third reason is that after being disturbed by human machinery and planting fast-growing plants, the mulch on the soil is reduced. The erosion intensity of rainfall on the soil increases, resulting in the loss of soil quality.

Soil pH and EC decreased significantly during the first five years of recovery. This could be because in the early stages of recovery, the soil cover is reduced, and a large amount of rainwater washes away the excess salt in the soil (Zeng et al., 2020). Or because after being transformed into an artificial ecological corridor, the amount of fertilizers artificially applied to the mollisols is reduced. Our research shows that the forest-river ecological corridor can quickly restore the mollisols cultivated land with high salinity level after years of cultivation and reach the pH value of regular mollisols after the natural restoration of about 5 years. Some studies have shown that increasing the biodiversity of fallow land can significantly increase the content of TP in the soil (Chmolowska et al., 2016). This study found that the total phosphorus content in the soil increased substantially during the first five years of the restoration of cultivated land into ecological corridors and remained stable at higher levels after that. We think a possible reason could be that a large amount of phosphate fertilizer was artificially input into mollisols every year during the cultivated period. Yet, most of it was lost or infiltrated with rainwater, and the phosphorus element that stayed in the soil was minor. The persistent phosphorus release capacity of cultivated soil is poor (Zhang et al., 2019). Another reason could be that after being restored to an ecological corridor, the species and quantity of organisms have been greatly improved compared with cultivated land. Among them, animal carcasses and feces are essential sources of soil phosphorus, and the phosphorus content in mollisols increases with the increase of animals in the corridor (Zeng et al., 2020). Our research shows that after restoration to ecological corridors, the phosphorus element in the soil increases significantly, and the soil quality is enhanced. In addition, the increase of phosphorus in the soil helps reduce phosphorus discharged into rivers with rainwater and alleviates eutrophication of adjacent water bodies.

A limitation of this study that we acknowledge is when the distance factor is between 30-80m and the recovery period is between 15-20 years, there are fewer samples, resulting in a significant difference between the collected results and the previous data. In subsequent studies, we can study the reasons for the changes in this segment.

### Protection width of ecological corridor based on mollisols protection

Based on the soil TN, SOM, and CEC indices that have the most significant influence on the distance-width axis in the RDA analysis, we believe that the unilateral protection width of the ecological corridor should be 125-150 m. This width has a good recovery effect and a high-cost performance; it can restore mollisols quality and provide habitat for some fauna. It also promotes botanical supplementation (Li et al., 2012). As other research on reforestation in Australia and elsewhere has shown, this system appears to have the lowest economic recovery costs (Kanowski et al., 2008). The relative effectiveness of mollisols quality restoration on cultivated lands through ecological corridors depends on their ability to restore ecosystem properties and their relative costs (Paul et al., 2010). In addition to the construction cost of the corridor itself, the land acquisition and protection of the mollisols cultivated land in the ecological corridor and the compensation to the farmers will also incur related costs. At present, some studies based on biodiversity conservation have delineated a unilateral ecological corridor with a width of 200-400m (Tang et al., 2021). However, our observations found that the ecological corridor based on the restoration of mollisols quality may only need a width of about 125-150 m on one side. Once the forest-river ecological corridor reaches a certain width, if the width of the corridor is increased, the growth rate of biodiversity will decrease.

In addition, the central river channel with high biodiversity will also reduce the radiation effect on the margin, and the corresponding increase in the quality of mollisols will also slow down. However, if the ecological corridor is too narrow, it will also harm the protection of mollisols. For example, we found that the soil in the range of 0-10m was affected by factors such as the rising tide in the rainy season and the erosion of the river. The measured soil quality value in this section was relatively low. Studies in other regions also support such observations (Cheng et al., 2021). Conversely, a smaller corridor width is not conducive to supporting large birds and mammals (Kanowski et al., 2008). We found that, based on the same restoration period, within the width range of 125-150 m, the growth rate of the TN indices was the fastest in the entire observation period, and TN could recover to the level of cultivated land outside the corridor where fertilizers were applied. The growth rate of the SOM indices is the fastest in the entire observation range, and the recovery value reaches and far exceeds the reference value of mollisols and exceeds the average value of cultivated land by 27.3%. The CEC indices reached the reference value of mollisols, reaching 80% of the average value of cultivated land outside the corridor. We found that the recovery of both TN and SOM metrics slowed after the protection width exceeded 130 m. After the protection width reached 200m, the SOM tended to increase for the better, but the cost of protection increased significantly. As for CEC indices, when the protection width exceeded 150m, the impact of the ecological corridor on the CEC decreased. In conclusion, we recommend 125-150 m as the unilateral width for ecological corridor restoration.

### Recovery time of ecological corridors based on mollisols protection

Based on the soil SOC, pH, and EC indices that have the most significant impact on the recovery timeline in the RDA analysis, the protection period of the ecological corridor should be no less than 13 years. During this period, the recovery effect and cost performance are relatively high. The balance between cost and recovery effect also applies to the choice of recovery time. In the ecological corridor, after the recovery time reached a certain level, the improvement will be stabalised, and the increase of biodiversity will be slowed down (Lenoir et al., 2021). The corresponding improvement in soil quality will also slow down, but the cost of conservation will increase significantly. However, the protection period of corridors is too short, which may not be enough to support the proliferation and habitation of large wild animals in corridors (Cheng et al., 2021).

In observation, the improvement of quality of mollisols in the ecological corridor was a relatively long period. Based on the same distance width, during the 13-year recovery, the growth rate of the SOC indices was the fastest in the entire observation period, and the SOC reached 80.4% of the fertilized cultivated land outside the corridor. The pH indices decreased from the high level of cultivated land to the standard reference value range of mollisols, and the decrease rate reached 16%. The EC indices decreased from the high level of cultivated land to within the normal reference value range of mollisols, a decrease of 68.1%. However, we found that after a recovery time of more than 13 years, the recovery of all three indices slowed down. Finally, after the recovery time of 30 years, the three indices showed an upward trend. However, the cost of protection increases substantially over a long time. Therefore, we recommend 13 years as the shortest period for ecological corridor protection.

Nevertheless, we also found that in summarizing the best recovery time, the model regression had fewer sampling points in the middle recovery time. The existing sample points were concentrated at the two ends with shorter and longer recovery times. It may be related to local policies. Local farmers restored some sections, and the recovery time of those sections was longer. However, as far as Harbin is concerned, the local government started to actively restore the Ashi River ecological corridor between 2010-2013. That section of the restored artificial corridor had a short recovery time. We acknowledge this as a study limitation, with a relatively small sample size for 15-25 years of recovery time.

## Conclusion

This research investigated the relationship between the stoichiometric ecological characteristics of mollisols and ecological corridor construction factors using the case study of Harbin. The research results show that ecological corridors can restore mollisols’ quality. The research results demonstrate that when restoring mollisols through ecological corridors, the quality of mollisols first decreases, then increases, and finally reaches and exceeds the reference value of standard mollisols. According to the TN, SOM, and CEC indices of mollisols, we should keep the recommended unilateral width at 125-150 m. According to the SOC, pH, and EC indices of mollisols, it is suggested that the ecological corridor should complete a natural restoration cycle of at least 13 years. As the width of the ecological corridor further increases, the TN and CEC indices tend to be stable, fluctuating around the normal value. After remaining stable for a while, the SOM index reappeared in an upward trend. With the further increase in the recovery time of the ecological corridor, the EC index stabilized near the normal value, and the pH index showed a trend of weak acidity, the SOC index has an upward trend. The next step is to obtain a more diverse sample of typical mollisols regions in Northeast China.

There is insufficient research on large-scale ecological restoration in typical mollisols areas in Northeast China, future research should comprehensively examine the restoration effects of ecological corridors in different areas in typical mollisols areas under different construction factors, such as Changchun and Suihua. This study provides insight into the ecological restoration of mollisols by ecological corridors and contributes to the existing academic literature from a mollisols ecological restoration perspective. This study is of great significance for the current stage of construction, delimitation and management of ecological corridors around large cities in typical mollisols regions in Northeast China, and contributes to ensuring the healthy operation of mollisols ecosystems in the area.

## Data availability statement

The raw data supporting the conclusions of this article will be made available by the authors, without undue reservation.

## Author contributions

Conceptualization, HX. Methodology, HX and SW. Software, HX. Validation, HX and SW. Formal analysis, JD. Investigation, HX. Resources, SW. Data curation, HX. Writing—original draft preparation, HX. Writing—review and editing, SW and JD. Visualization, HX. Supervision, SW and JD. Project administration, SW. Funding acquisition, SW. All authors contributed to the article and approved the submitted version.

## Funding

This study was sponsored by the general-time and professional integration curriculum and teaching material system in the field of land and space planning, the second batch of new engineering research and practice projects of the Chinese Ministry of Education (E-ZYJG20200215). This study was sponsored by Research on green campus and surrounding space planning based on sustainable development (XNAUEA5750000120). Financial support from the program of China Scholarships Council (No. 202106120248).

## Acknowledgments

We thank Liu yan in Heilongjiang Academic of Environmental Science and Bai xin in Harbin Ecological Environment Monitoring Center of Heilongjiang Province for helping with this experiment. At the same time, we would like to thank Brother Yu Hong and Brother Zhao Jindong for their help in the experimental sampling part.

## Conflict of interest

The authors declare that the research was conducted in the absence of any commercial or financial relationships that could be construed as a potential conflict of interest.

## Publisher’s note

All claims expressed in this article are solely those of the authors and do not necessarily represent those of their affiliated organizations, or those of the publisher, the editors and the reviewers. Any product that may be evaluated in this article, or claim that may be made by its manufacturer, is not guaranteed or endorsed by the publisher.
